# Scatter and Blurring Compensation in Inhomogeneous Media Using a Postprocessing Method

**DOI:** 10.1155/2008/806705

**Published:** 2009-03-04

**Authors:** Yan Yan, Gengsheng L. Zeng

**Affiliations:** Department of Radiology, Utah Center for Advanced Imaging Research, University of Utah, 729 Arapeen Drive, Salt Lake City, UT 84108, USA

## Abstract

An efficient postprocessing method to compensate for the scattering and blurring effects in inhomogeneous medium in SPECT is proposed. A two-dimensional point spread function (2D-PSF) was estimated in the image domain to model the combination of these two physical effects. This 2D-PSF in the inhomogeneous medium is fitted with an asymmetric Gaussian function based on Monte Carlo simulation results. An efficient further blurring and deconvolution method was used to restore images from the spatially variant 2D-PSF kernel. The compensation is performed using a computer-simulated NCAT phantom and a flanged Jaszczak experimental phantom. The preliminary results demonstrate an improvement in image quality and quantity accuracy with increased image contrast (25% increase compared to uncompensated image) and decreased error (40% decrease compared to uncompensated image). This method also offers an alternative to compensate for scatter and blurring in a more time efficient manner compared to the popular iterative methods. The execution time for this efficient postprocessing method is only a few minutes, which is within the clinically acceptable range.

## 1. INTRODUCTION

Single photon emission computed tomography (SPECT) images are degraded by attenuation, collimator and detector blurring, and photon scatter. Several studies have shown that compensations for these degradations can improve the quantitative accuracy and clinical lesion detectability [1–6]. The goal of this study is to develop a new method that can compensate for the scatter and blurring effects and improve the quantitative and qualitative accuracy of clinically realistic SPECT images. Currently, the state-of-the-art compensation method is to model the scatter and blurring effects in the projector/backprojector pair of an iterative reconstruction algorithm [7–20]. The main problem with this iterative compensation method is its heavy computational burden. Also, preprocessing procedures have been investigated to compensate for these physical degradations. The blurring is compensated in a preprocessing procedure such as using the frequency-distance principle [21–24]. The scatter is corrected using energy-distribution-based methods [25–27].

Previously, we have proposed a postprocessing method to compensate for the scattering in homogeneous media [28]. In this paper, we extend the postprocessing method to compensate for the scatter and blurring in inhomogeneous scattering media. We first reconstruct a raw image using an efficient analytical or iterative algorithm that corrects for attenuation only. We then model the scatter and blurring using a spatially variant two-dimensional point spread function (2D-PSF) in the inhomogeneous scattering media and parameterize the 2D-PSF based on Monte Carlo simulations. Finally, we use an efficient further blurring and deconvolution method to restore the image.

## 2. METHOD

### 2.1. Monte Carlo simulations

Monte Carlo simulations have been widely used in different areas of medical physics with the advantage of powerful computing systems [29, 30]. These Monte Carlo modeling techniques are ideal for SPECT because of the stochastic nature of radiation emission, transport, and detection processes. However, they require very long computational times. In this paper, we used the Monte Carlo simulation package SIMSET [31] to generate SPECT data with scatter contamination and detector response. The Monte Carlo data was used as a standard for scatter and blurring modeling. In the simulation, the collimator was modeled as a parallel hole collimator with a thickness of 2 cm and hole diameter of 0.14 cm. The detection energy window was centered at 140 keV with a width of 10%. The radius of rotation was 20 cm. For each phantom study, two sets of projection data were simulated. The first dataset was primary photons representing an ideal data acquisition, in which scattered photons were perfectly rejected. The second dataset contained scattered photons only. In each simulation, one billion photon histories were generated to yield low-noise projection data.

### 2.2. Computer simulation phantom

**Figure 1:**
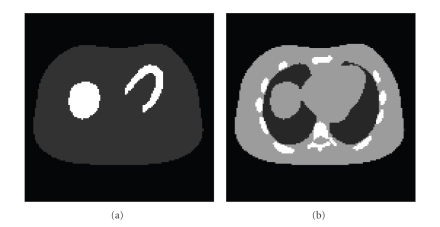
Two-dimensional NCAT phantoms: (a) activity distribution; (b) nonuniform attenuation distribution.

An NCAT phantom [32] was used in computer simulations. The attenuation map and activity distribution are shown in Figure 1. The intensity ratio of the activity in the myocardium versus background tissues was 5:1. The 40 cm × 40 cm object region was digitized onto a 129 × 129 array with a pixel size of 0.31 cm (the array size of 129 × 129 was chosen to allow the placement of the point source in the center of the object). The object is centered on the SPECT camera's rotation axis. The projection data was collected with 300 view angles over a full 360°.

### 2.3. Experimental phantom

A flanged Jaszczak hot-rod/cold-sphere phantom was scanned for one hour using a Philips IRIX SPECT system. The phantom was filled with water and 21.6 mCi of Tc-99m. Three low-energy high-resolution parallel-hole collimators were used during data acquisition. The rotation radius of the collimators was 24 cm. The data were collected with 180 view angles over a full 360°. The image was reconstructed in a 128 × 128 array with an image pixel size of 0.28 cm.

### 2.4. The postprocessing method

Our postprocessing method consists of three steps: (1) an efficient analytical or iterative algorithm that corrects for attenuation only is used to reconstruct a raw image; (2) a spatially variant two-dimensional point spread function (2D-PSF) in the inhomogeneous scattering medium is estimated; (3) an efficient, noniterative method is developed to restore the image.

#### 2.4.1. Reconstruction algorithm

We started with the raw SPECT projection data. They were contaminated by attenuation, scattering, and collimator blurring. Instead of trying to subtract the estimated scattered data from the projections, we can directly reconstruct the raw image from these projection data using either an analytical reconstruction algorithm [33–36] or an iterative ML-EM reconstruction algorithm [37–39]. Here, we used the iterative algorithm as follows:

(1) f^(xi)new=f^(xi)old∑jaij∑jaijpj∑kakjf^(xk)old,

where *x*_*i*_ represents one pixel in the image space, *p*_*j*_ is the measured SPECT emission data, and *a*_*i j*_ is the known coefficient that represents the contribution of image pixel *i* to projection bin *j* with the attenuation map *μ*. The summation over *k* is the projector, and the summation over *j* is the backprojector. This algorithm reconstructs a raw image $\hat{f}$ with attenuation compensation (AC). However, the scattering and collimator blurring are not corrected. Our postprocessing method was applied to this raw image $\hat{f}.$

#### 2.4.2. The two-dimensional point spread function (2D-PSF)

The raw image $\hat{f}$ can be modeled as a blurred version of the original image *f *:

(2) f^(x0,y0) =∫x=−ΔΔ ∫y=−ΔΔh(x0,y0;x,y)f(x0−x,y0−y)dx dy,

where the blurring kernel *h*(*x*_0_, *y*_0_; *x*, *y*) is what we call a 2D-PSF in the image domain, and Δ is a small positive number (i.e., Δ = 7 in our study), representing half the size of the kernel *h*. The discrete version of this relation can be written as

(3) f^(i,j)=∑l=−ΔΔ ∑m=−ΔΔh(i,j;l,m)f(i−l,j−m).

For each image pixel (*i*, *j*), *h* is a 2D blurring matrix with the size of (2Δ + 1) × (2Δ + 1). The true image *f* can be solved if the kernel *h* is known. However, this 2D-PSF *h* contains the effects of collimator blurring and scattering and it is normally hard to obtain. Furthermore, the 2D-PSF is spatially variant, which means that it changes for every image pixel (*i*, *j*).

This 2D-PSF models the scattering effect and collimator blurring in the 2D image domain instead of in the conventional 1D projection domain. It is also different from the “effective scatter source image” as proposed by Frey and Tsui [11]. Both being in the image domain, the effective scatter source image is different for each projection view and when a projection is applied to this effective image, the estimated scattered projection at this view is obtained; our proposed 2D-PSF is a kernel that relates the true image and the raw reconstructed image. Also, we can obtain any projected blurring kernel by performing an attenuated projection operator on the 2D-PSF.

We model the 2D-PSF *h* in (3) as a Gaussian function with five variables (a short explanation of the reason why we are able to use the simple Gaussian function is discussed in the appendix): the magnitude of the Gaussian *A*_0_, full width at half-maximum in the long-axis direction FWHM_*l*_, full width at half-maximum in the short-axis direction FWHM_*s*_, and the center (*x*_0_, *y*_0_) of the Gaussian:

(4) $$\begin{array}{ll} & h(x_{0},y_{0};x,y)\\ & \;=A_{0}\;\;\exp\;(-\frac{4\ln\;(2){\left(x-x_{0}\right)}^{2}}{{\mathrm{FWHM}}_{l}^{2}}-\frac{4\;\;\ln\;(2){\left(y-y_{0}\right)}^{2}}{{\mathrm{FWHM}}_{s}^{2}}), \end{array}$$

where *A*_0_, FWHM_*l*_ and FWHM_*s*_ are the functions of the point source position (*x*_0_, *y*_0_).

**Figure 2:**
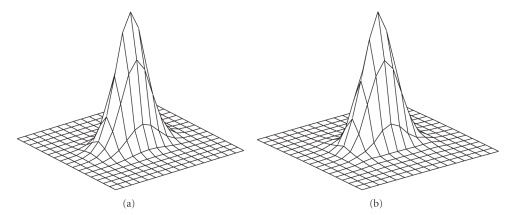
Comparison of a 2D-PSF of a point source located at a distance of 7.5 cm from the rotation center using the Monte Carlo simulation and the Gaussian model; (a) Monte Carlo simulation; (b) Gaussian model.

Here, we demonstrate a similarity comparison of a measured 2D-PSF and its corresponding Gaussian fitting function. Figure 2(a) is the 2D-PSF calculated from a point source at a distance of 7.5 cm to the center of the rotation using Monte Carlo simulation. Figure 2(b) is the Gaussian function with parameters fitted to the 2D-PSF. The comparison indicates that the two-dimensional Gaussian distribution is a good fit for the 2D-PSF.

#### 2.4.3. Parameterization of 2D-PSF

**Figure 3:**
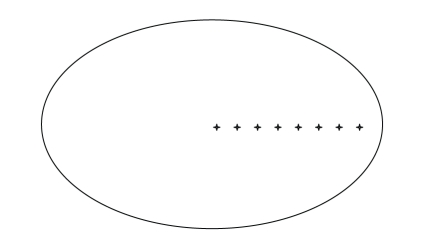
Estimation of the image domain 2D-PSF *h* by performing Monte Carlo simulations for eight different point source locations (marked by plus signs).

In order to observe the variations of the 2D-PSF in different locations of the object, we perform Monte Carlo simulations for point source at eight different locations. A uniform cylinder phantom with elliptical cross-sections is used. The raw reconstructed image of each point source is related to the 2D-PSF at the same location. The locations of the point sources are displayed in Figure 3.

**Figure 4:**
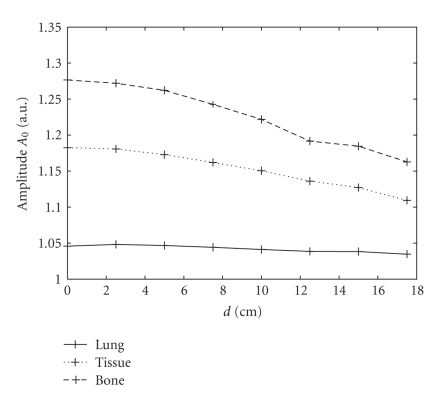
Variations in amplitude of the 2D-PSF as a function of *d* from eight-point Monte Carlo simulations. Solid, dotted, and dashed lines represent the simulation using a uniform attenuation map with coefficients of 0.04 cm^−1^ (lung), 0.15 cm^−1^ (soft tissue), and 0.25 cm^−1^ (bone), respectively.

**Figure 5:**
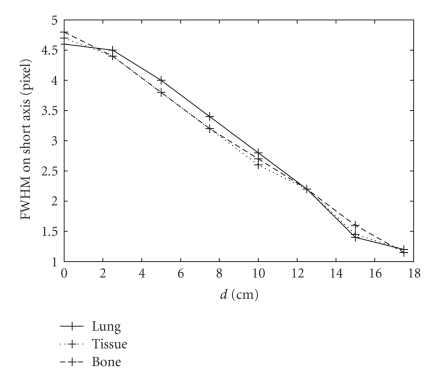
Variations in FWHM on the short axis of the 2D-PSF as a function of d from eight-point Monte Carlo simulations. Solid, dotted, and dashed lines represent the simulation using a uniform attenuation map with coefficients of 0.04 cm^−1^ (lung), 0.15 cm^−1^ (soft tissue), and 0.25 cm^−1^ (bone), respectively.

**Figure 6:**
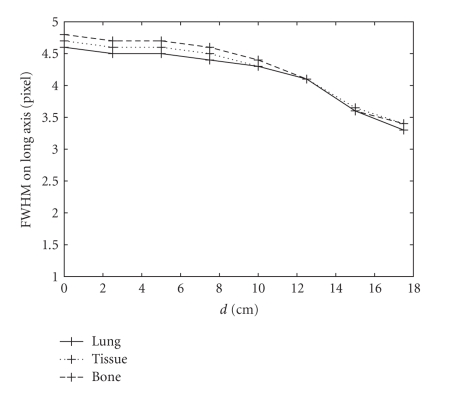
Variations in FWHM on the long axis of the 2D-PSF as a function of d from eight-point Monte Carlo simulations. Solid, dotted, and dashed lines represent the simulation using a uniform attenuation map with coefficients of 0.04 cm^−1^ (lung), 0.15 cm^−1^ (soft tissue), and 0.25 cm^−1^ (bone), respectively.

In our previous study of the 2D-PSF [40], we discovered that in homogeneous scattering media, the 2D-PSF is rotationally symmetric with respect to the rotation center, which means that the 2D-PSF with a constant radial distance has the same shape for all angles but is rotated by a certain angle. Therefore, the 2D-PSF is estimated only on the positive *x*-axis (Figure 3). Also, because of the localized character (i.e., small width) of the 2D-PSF, it is convenient to have this assumption in the inhomogeneous case except for the variations from the different attenuation coefficients. For rotationally symmetric point sources in inhomogeneous media, if the attenuation coefficients are the same for two different point locations, we assume that the 2D-PSFs are the same; if the attenuation coefficients are different, we estimate the 2D-PSF according to different attenuation factors. To estimate the variations of the 2D-PSF with local attenuation coefficients, three sets of Monte Carlo simulations have been performed. In each of these three simulations, we use uniform attenuation maps with the same shapes but with different coefficients: *μ*_1_ = 0.25 cm^−1^ representing the bone, *μ*_2_ = 0.15 cm^−1^ representing the tissue, and *μ*_3_ = 0.04 cm^−1^ representing the lung. All the other configurations are the same for these three simulations. In Figures 4–6, we show the variations of the amplitude *A*_0_, FWHM on the short axis, and FWHM on the long axis as a function of *d*, the distance from the point source to the rotation center. The variations of the amplitude *A*_0_ are shown in Figure 4. It decreases with *d* and also varies for different attenuators. The values of *A*_0_ are larger in highly attenuated objects: largest in the bone and smallest in the lung. This distribution agrees with the scatter probability derived from the Klein-Nishima formula [41]. We fit *A*_0_ as a function of both *d* and the attenuation distribution *μ*:

(5) $$A_{0}(d,\mu )=1.18P_{1}(\mu )-2.5\times 10^{-3}d\cdot P_{2}(\mu ),$$

in which

(6) $$\begin{array}{ll} P_{1}(\mu ) & =0.85+1.14\;\mu -0.73\;\mu^{2},\\ P_{2}(\mu ) & =0.16+4.06\;\mu +18.46\;\mu^{2}, \end{array}$$

where *A*_0_ is dimensionless and represents the relative magnitude increase due to scattering. The second-order polynomial of the attenuation factors is chosen to get reasonably well-fitting results.

The full width at half-maximum (FWHM) of the Gaussian function is determined by the combined effects of collimator blurring and scatter blurring. Because the amplitude of the reconstructed image from the scattered data is small compared to the reconstructed image from the primary photons (see the appendix), the FWHM of the *h* is mainly determined by the FWHM of the reconstructed image from the primary photons. Therefore, the blurring in the 2D-PSF is most affected by collimator blurring and is independent of the attenuators. This is verified by the simulation results as shown in Figures 5 and 6. As the source moves from the center of the object toward the edge, the value of FWHM_*s*_ decreases (Figure 5). Little change is observed among three different objects. Figure 6 shows a plot of FWHM_*l*_, the full width at half-maximum on the long axis of the Gaussian function. It is observed that the value of FWHM_*l*_ barely changes as the source moves from the center toward the edge. Also, note that the largest difference is a little over one pixel. Similar variations are proposed by Zeng and Huang [42]. These two parameters are fitted based on a distance-dependent model [43], which will not change for different attenuators:

(7) $$\begin{array}{ll} {\mathrm{FWHM}}_{l}(d) & =2r+\frac{2r\times D}{l},\\ {\mathrm{FWHM}}_{s}(d) & =2r+\frac{2r\times (D-d)}{l}, \end{array}$$

where *r* is the radius of the collimator hole, *l* is the thickness of the collimator, *d* is the distance from the point source to the center of the object *,* and *D* represents the distance from the center of rotation to the detector in cm, which equals to 20 cm in our simulations.

With (4)–(7), we can calculate the 2D-PSF for any point source inside the object. These empirical formulae eliminate the need for extensive Monte Carlo simulations for each point source. Also, the estimation of the 2D-PSF using these formulae is independent of the raw image of the simulation phantom. It is derivable from the attenuation map and configurations of the collimator and can be calculated before reconstruction.

#### 2.4.4. Image restoration

As the 2D-PSF is a spatially variant, a normal deconvolution algorithm cannot be used. In order to avoid the long computational time of using iterative restoration algorithms, we used a further-blurring and deconvolution method to restore the image [42]. This method converted the raw image with a spatially variant point spread function into a further blurred image with a spatially invariant point spread function, and then used an efficient technique (e.g., a frequency domain filtering) for deblurring.

The further blurring was implemented by using a rotational convolution. Let the raw reconstructed image be f *. We rotated the image f * counterclockwise by a small angle *θ* about the axis of the detector rotation obtaining *f*_θ_* and rotated f * clockwise by *θ* obtaining *f*_−θ_*. When necessary, we rotated the image f * counterclockwise by 2*θ* obtaining *f*_2θ_* and rotated f * clockwise by 2*θ* obtaining *f*_−2θ_* and so on. A weighted sum of these rotated images gives a further blurred image $\hat{G}:$

(8) G^=1A0(d)∑nanfnθ*, n=0,±1,±2,±3,…,

where the weighting factors *a*_*n*_ form a convolution kernel. The sum of *a*_*n*_ is normalized to 1 to assure the consistency of the image intensity. The weighting factors *a*_*n*_ are chosen empirically, so that the 2D-PSF is spatially invariant. *A*_0_ is the amplitude of 2D-PSF discussed in Section 3, which is used to normalize the amplitude of the raw image f * with different radial distances *d*.

Now, this further blurred image has an approximately spatially invariant point spread function *h*_0_ and this *h*_0_ is nothing but the 2D-PSF at the center of the object. Then, we perform an efficient inverse filtering (e.g., the Wiener Filtering) on this image $\hat{G}$ to obtain the restored image *f* in (1).

### 2.5. Assessment of restored images

Several measurements were performed in the computer simulation results to evaluate the improvement of the image quality using the proposed compensation method.

- Sum-squared error (SSE) was used to measure the average discrepancy of the restored image with respect to the original image. It is defined as the averaged sum of the squared pixel difference as follows:
  (9) SSE=1N∑i=1N(f^(xi)−f(xi))2,
  where $\hat{f}(x_{i})$ and *f *(*x*_*i*_) represent the restored value and the true value for pixel *i*, respectively, and *N* is the total number of pixels calculated.
- Contrast (CR) between myocardium and background is defined as
  (10) $$\text{CR}=\frac{|FG-BG|}{FG+BG},$$
  where *FG* represents the average pixel value in the myocardium, and *BG* represents the average pixel value in the background.
- Noise was measured as the standard deviation of pixel counts in the uniform background, normalized by the mean activity of that region.

## 3. RESULTS

### 3.1. Computer simulations

**Figure 7:**
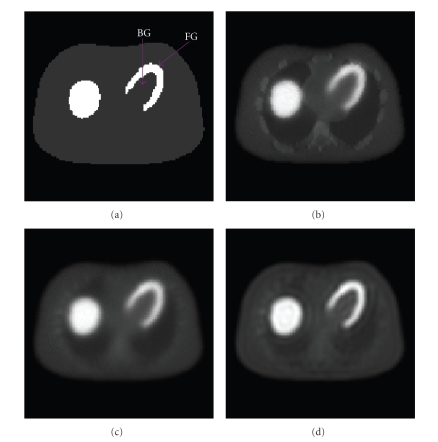
(a) NCAT phantom image, the regions of interest for contrast analysis are marked; (b) raw reconstructed image without using blurred attenuation map; (c) raw reconstructed image using blurred attenuation map; (d) restored image using the proposed method.

**Figure 8:**
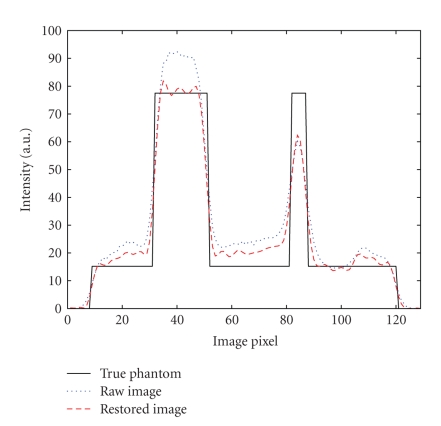
Horizontal profiles through the center of the images.

The Monte Carlo simulation data for the NCAT phantom was used to reconstruct the raw image using the ML-EM iterative algorithm. The attenuation correction was performed in the ML-EM reconstruction using a blurred attenuation map. The map was blurred with a Gaussian function to match the resolution of the emission data. The parameters of this Gaussian function are empirically chosen to obtain the optimal reconstructed image with least cross-talk artifacts. Figure 7(b) demonstrates the raw reconstructed image without using the blurred attenuation map, in which there were cross-talk effects due to the unmatched resolution between the attenuation map and the emission data. Figure 7(c) shows the raw image reconstructed using the blurred attenuation map. The 2D-PSF was then estimated using a smeared attenuation map and collimator parameters. This smeared attenuation map used in the 2D-PSF estimation is different from the blurred map used in the reconstruction. This smearing is to integrate the influence of neighborhood pixels into the point source because the 2D-PSF represents the total scattered photons that originate from a point source and interact with its neighborhood pixels. For restoration, the further blurred image $\hat{G}$ was obtained by rotational convolution:

(11) G^=1A0{0.2f*+0.16(f1°*+f−1°*)+0.12(f2°*+f−2°*)+0.08(f3°*+f−3°*)+0.04(f4°*+f−4°*)},

where f * represents the raw image, and *f*_θ_* represents the image rotated by an angle *θ*. The weighting factors were determined empirically to get image $\hat{G},$ so that it has a spatially invariant point spread function. Wiener filtering was then performed to restore the further blurred image $\hat{G}.$ Figure 7(d) shows the restored image. It is observed that there exists a dishing effect in the liver and boundary area. This is caused by the over filtering in the inverse filtering step. The tradeoff between over filtering and the effectiveness of the inverse filtering is a limitation of our method and needs further investigation in the future. A horizontal profile through the center of the images is shown in Figure 8.

The contrast, SSE, and noise were calculated for all images to illustrate the improvement of image quality in the restored image (Table 1). The raw image here is reconstructed with a blurred attenuation map. It is observed that after compensation, the quantitative accuracy and contrast were improved, and noise was controlled from being elevated.

### 3.2. Phantom experiment

The ML-EM iterative algorithm with 50 iterations was used for raw image reconstruction and attenuation correction. The 2D-PSF was estimated based on the water-filled uniform attenuator and the low-energy high-resolution collimator. For image restoration, the further blurred image $\hat{G}$ was obtained by rotational convolution as follows:

(12) G^=1A0{0.4f*+0.2(f1°*+f−1°*)+0.1(f2°*+f−2°*)}.

**Figure 9:**
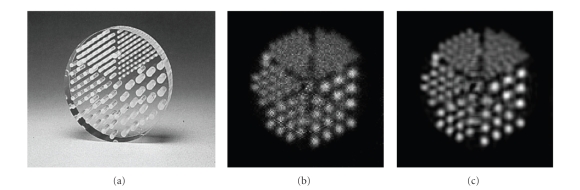
(a) Jaszczak phantom; (b) raw reconstructed image; (c) restored image using the proposed method.

The restored image was obtained after application of Wiener filtering. Figure 9(c) shows the restored image. It is observed that the restored image is less noisy than the raw image, as shown in Figure 9(c) compared to Figure 9(b). This may be due to the fact that the Wiener filter is a band-pass filter, and the high-frequency noise is suppressed.

## 4. DISCUSSIONS

The goal of this postprocessing method is to develop a time efficient compensation method and overcome the heavy computational burden in the iterative reconstruction-based method. There are several issues to mention in this section.

### 4.1. Accuracy and generality of the 2D-PSF estimation

The estimation of the 2D-PSF is the main challenge of the proposed method. The 2D-PSF models the scattering and collimator blurring in the image domain instead of the conventional projection domain. We used a Gaussian function to approximately model the 2D-PSF. The validity of using the Gaussian function was discussed. We derived empirical formulae for the parameters of the Gaussian function from the Monte Carlo simulations. As the 2D-PSF is object-dependent, we need to pay attenuation to the generality of the estimations. As discussed in this study, the parameter *A*_0_ of the Gaussian function depends on the local attenuation coefficient, and the FWHMs stay the same for different attenuation distributions and only depend on collimator configurations. More Monte Carlo simulations for objects with various sizes and shapes are desired to further determine a more accurate and general 2D-PSF model.

### 4.2. “Further blurring and deconvolution” restoration

This method efficiently restores images with spatially variant point spread functions. The advantage of this approach is its fast implementation compared with the conventional iterative algorithms. However, this is an approximate method, and the rotation angle *θ* and the weighting factors in (8) are currently determined empirically. As the goal of the postprocessing method is to cut down the computational time for the compensation, an efficient restoration method like this one is desired. Other efficient restoration methods with the capability of deblurring spatially variant point spread functions can also be used.

### 4.3. Computational time

The computational time for this postprocessing compensation method was reduced compared to the iterative reconstruction-based method. The current time for fast implementation of the reconstruction-based method for a 64 × 64 × 64 image array is in the range of thirty minutes [1, 17]. In this proposed postprocessing method, the computer time for getting a two-dimensional attenuation-corrected raw image was in the order of seconds for fast reconstruction algorithms [34, 35, 39]. We then precalculated the 2D-PSF and stored it in the computer memory. In the last step, all we needed was a few more seconds for image restoration (i.e., rotational convolution and Wiener filtering). Therefore, the total computer time for this postprocessing compensation method was only few minutes and is acceptable for clinical applications.

## 5. CONCLUSIONS

We have presented an efficient postprocessing method to compensate for scattering and blurring effects in inhomogeneous media. The major challenge of the method is to accurately estimate the 2D-PSF in the image domain. Empirical formulae are proposed to model the 2D-PSF variations with the various locations within nonuniformly attenuated objects. From the clinical aspect, the implementation of our method is faster (within several minutes) than the iterative reconstruction-based compensation method. One limitation of this study is that it is developed in two dimensions and does not consider scattered photons from out-of-plane sources. Our future work includes modeling the scattering with a 3D-PSF.

## Figures and Tables

**Figure 10:**
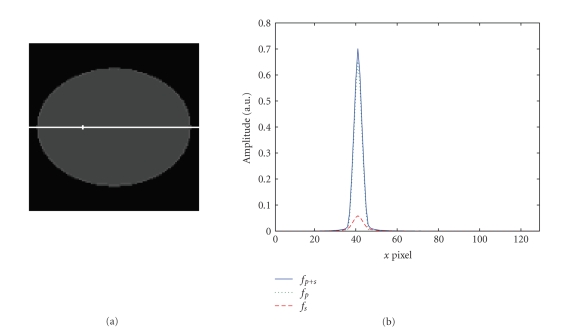
Profile of the raw images along the major axis. Solid line: image *f*_*p*+*s*_; dotted line: image *f*_*p*_; dashed line: image * f*_*s*_.

**Figure 11:**
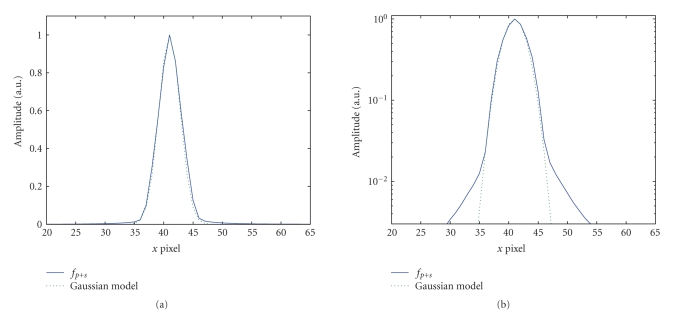
Comparison of the 2D-PSF and the fitted Gaussian function. Profiles are plotted along the long axis direction. Solid line: 2D-PSF (image *f*_*p*+*s*_); dotted line: Gaussian fitted function. (a) Linear scale; (b) logarithmic scale.

**Figure 12:**
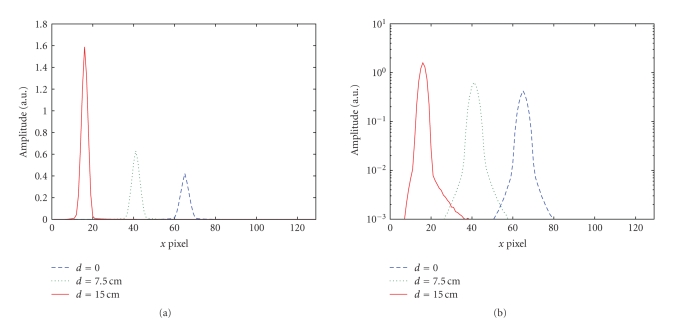
Profile of the 2D-PSF on the long axis for point sources with three different radial distances. Dashed line: radial distance *d* = 0; dotted line: radial distance *d* = 7.5 cm; solid line: radial distance *d* = 15 cm. (a) Linear scale; (b) logarithmic scale.

**Table 1:** Comparisons of image quality for computer simulation results.

	True image	Raw image	Restored image	Improvement
Sum-squared error	0	40	24	−40%
Contrast	0.66	0.39	0.50	+25%
Noise	0	0.011	0.014	+27%

## APPENDIX

Monte Carlo simulations are performed to generate two sets of projection data: one is the primary scatter-free data, denoted by *p*, and the other is the scattered data only, denoted by *s*. Both datasets are contaminated by blurring effect. We use *f*_*p*_ and *f*_*s*_ to represent the raw reconstructed images from primary and scattered photons, respectively. The sum of these two images (denoted by *f*_*p*+*s*_) is the raw image with both scatter and blurring contaminations. As defined in (1), the amplitude of the 2D-PSF is determined by the total volume change in the image *f*_*p*+*s*_ with repect to *f*_*p*_ Figure 10 shows the profiles of the *f*_*p*_, *f*_*s*_ and *f*_*p*+*s*_ of a point source. Although the ratio of total volume from the image *f*_*p*_ over the total volume from the image *f*_*s*_ is around 2:1, the amplitude of the *f*_*p*_ is much larger than that of the *f*_*s*_. Therefore, the shape of the 2D-PSF is determined by the shape of point source image *f*_*p*_. Also in Figures 11(a) and 11(b), we demonstrate the comparison of the 2D-PSF (related to the *f*_*p*+*s*_) with the fitted Gaussian function in both linear and logarithmic scales. It is observed that the Gaussian function fits the overall shape of the 2D-PSF very well except in the tails of the 2D-PSF. The discrepancy in the tails is magnified in Figure 11(b) in logarithmic scale. Compared with the amplitude and total area of *f*_*p*_, the discrepancy in the tails from the Gaussian fittings is very small and neglectable. Therefore, the Gaussian function is a good fit for the 2D-PSF.

Another point worth mentioning is the asymmetry shape along the long axis direction of the 2D-PSF. Previous study [9] discovered the asymmetric shape of the projection-domain scatter response function and modeled it with two different Gaussians on either side of the point source. In our approach, it is not necessary to model the scatter with two Gaussians. One reason for that is our 2D-PSF is a reconstructed image from all the asymmetric projections. The asymmetry in the projections is balanced out and not significant observed in the 2D-PSF. Figures 12(a) and 12(b) show the plots of the 2D-PSF on the long axis for point sources with three different radial distances. The asymmetry can be barely observed even in the logarithmic scale. Furthermore, as the amplitude of *f*_*s*_ is much smaller than that of *f*_*p*_ as observed in Figure 10, the asymmetric distribution of the scatter image *f*_*s*_ contributes little to the image *f*_*p*+*s*_, which represents the overall effects of both blurring and scatter. Therefore, it is reasonable to model the scatter and blurring with one Gaussian function in (4).
